# Activation of Nrf2 by the dengue virus causes an increase in CLEC5A, which enhances TNF-α production by mononuclear phagocytes

**DOI:** 10.1038/srep32000

**Published:** 2016-08-26

**Authors:** Yi-Lin Cheng, Yee-Shin Lin, Chia-Ling Chen, Tsung-Ting Tsai, Cheng-Chieh Tsai, Yan-Wei Wu, Yi-Dan Ou, Yu-Yi Chu, Ju-Ming Wang, Chia-Yi Yu, Chiou-Feng Lin

**Affiliations:** 1 Institute of Basic Medical Sciences, College of Medicine, National Cheng Kung University, Tainan 701, Taiwan; 2 Center of Infectious Disease and Signaling Research, National Cheng Kung University, Tainan 701, Taiwan; 3 Department of Microbiology and Immunology, College of Medicine, National Cheng Kung University, Tainan 701, Taiwan; 4 Translational Research Center, Taipei Medical University, Taipei 110, Taiwan; 5 Department of Microbiology and Immunology, College of Medicine, Taipei Medical University, Taipei 110, Taiwan; 6 Department of Nursing, Chung Hwa University of Medical Technology, Tainan 717, Taiwan; 7 Institute of Bioinformatics and Biosignal Transduction, National Cheng Kung University, Tainan 701, Taiwan; 8 Graduate Institute of Medical Sciences, College of Medicine, Taipei Medical University, Taipei 110, Taiwan

## Abstract

Infection by the dengue virus (DENV) threatens global public health due to its high prevalence and the lack of effective treatments. Host factors may contribute to the pathogenesis of DENV; herein, we investigated the role of nuclear factor (erythroid-derived 2)-like 2 (Nrf2), which is activated by DENV in mononuclear phagocytes. DENV infection selectively activates Nrf2 following nuclear translocation. Following endoplasmic reticular (ER) stress, protein kinase R-like ER kinase (PERK) facilitated Nrf2-mediated transcriptional activation of C-type lectin domain family 5, member A (CLEC5A) to increase CLEC5A expression. Signaling downstream of the Nrf2-CLEC5A interaction enhances Toll-like receptor 3 (TLR3)-independent tumor necrosis factor (TNF)-α production following DENV infection. Forced expression of the NS2B3 viral protein induces Nrf2 nuclear translocation/activation and CLEC5A expression which increases DENV-induced TNF-α production. Animal studies confirmed Nrf2-induced CLEC5A and TNF-α in brains of DENV-infected mice. These results demonstrate that DENV infection causes Nrf2-regulated TNF-α production by increasing levels of CLEC5A.

Dengue is an arthropod-borne viral disease which infects more than 390 million people annually. Owing to the increase in the number of cases, the expansion of geographic distribution, and disease severity, dengue has become a serious global public health issue^1^. The dengue virus (DENV) is an enveloped, positive-stranded RNA virus of the Flaviviridae family that is transmitted by *Aedes* mosquitoes^2^. It contains four serotypes and consists of three structural proteins including an envelope (E) protein, precursor membrane (prM) protein, and capsid (C) protein, as well as seven types of nonstructural (NS) proteins, including NS1, NS2A, NS2B, NS3, NS4A, NS4B, and NS5^3^. Although most people infected by DENV are asymptomatic, some develop severe disorders ranging from dengue fever (DF) to severe dengue diseases, which include a potentially lethal hemorrhagic and capillary leak syndrome, termed dengue hemorrhagic fever (DHF) and dengue shock syndrome (DSS), with multiple organ involvement^2^,^4^. Unfortunately, to date, there is no effective treatment, and a currently approved vaccine still needs further testing to verify its long-term efficacy.

The pathogenesis of severe dengue remains a challenging puzzle with many missing pieces. Multiple risk factors are believed to be involved, including virus virulence, antibody-dependent enhancement, T cell responses, complement activation, autoimmune responses, host factors, cytokine storms, and vascular leakage^5^,^6^. Elevated proinflammatory and vasoactive cytokines, which were correlated with the disease severity, are observed in patients with DHF and DSS before and at the time of plasma leakage^7^. Previous reports identified C-type lectin domain family 5, member A (CLEC5A), also known as myeloid DAP12-associating lectin, as a critical DENV signaling receptor responsible for inflammatory activation^8^. After neutralizing CLEC5A by monoclonal antibodies, the production of several proinflammatory cytokines, including tumor necrosis factor (TNF)-α, interleukin (IL)-6, IL-8, macrophage inflammatory protein-1α, and interferon (IFN)-inducible protein-10, are significantly attenuated^8^. In addition, activating CLEC5A in immature myeloid cells by DENV was also demonstrated to induce lethal shock through TNF-α and nitric oxide in mice^9^. However, regulation of CLEC5A expression during DENV infection is still largely unknown.

Host transcription factors (TFs), which can be translocated into nuclei and modulate transcription of different genes, must also be considered during DENV infection^10^. Multiple DENV NS proteins are reported to inhibit type I IFN production or signaling through blocking TFs IRF3, or STAT^11^. Moreover, DENV also activates several TFs, such as STAT3, p-TEFb, and nuclear factor (NF)-κB, to induce chemokine or cytokine production^12^,^13^,^14^. While no direct evidence exists to demonstrate the regulation of CLEC5A by DENV-activated TFs, it is speculated that CLEC5A is a potential target gene of nuclear factor (erythroid-derived 2)-like 2 (Nrf2). This is based on a study by Hirotsu *et al.* in which genome-wide screening was performed to identify Nrf2-binding sites through chromatin immunoprecipitation (ChIP) coupled with high-throughput sequencing under diethyl maleate treatment, an inducer of Nrf2^15^. Following screening of luciferase-based reporter arrays for 45 TFs, we investigated the molecular regulation and novel role of activated Nrf2 in DENV infection, particularly for CLEC5A-regulated TNF-α expression.

## Results

### DENV infection induces Nrf2 activation in mononuclear phagocytic cells.

**Figure 1:**
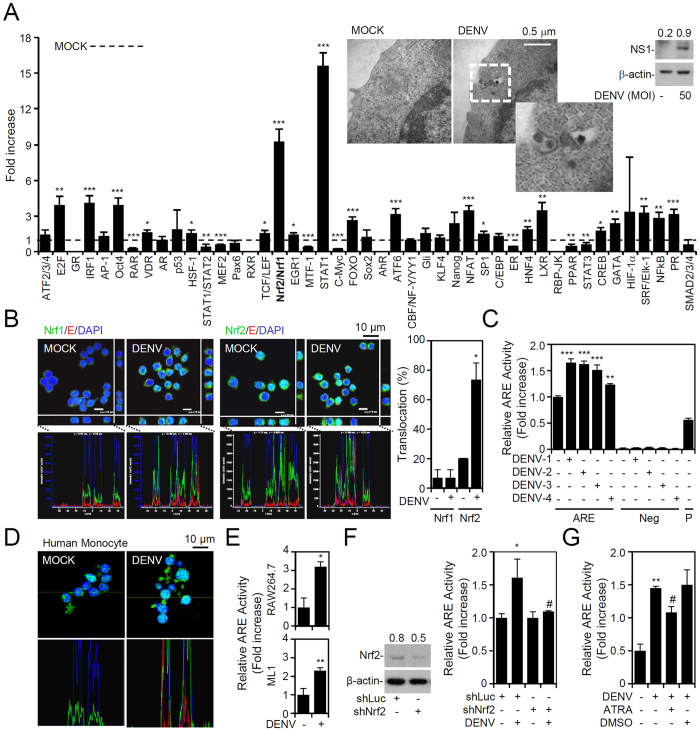
Dengue virus (DENV) infection induces nuclear factor (erythroid-derived 2)-like 2 (Nrf2) activation in phagocytic mononuclear cells. (**A**) DENV infection (6 h post-infection) in RAW264.7 cells was demonstrated by a Western blot analysis of viral NS1 expression and electronic microscopic images of virions. The reporter array monitored the activation of 45 transcription factors (TFs), which were quantified by measuring the luciferase activity. (**B**) Confocal immunostaining for nuclear translocation of Nrf1 and Nrf2 (*green*) in DENV-infected cells (E protein positive, *red*) was quantified and is shown as a percentage. DAPI was used for nuclear staining (*blue*). (**C**) The antioxidant response element (ARE) activity assay was performed to measure Nrf2 activation caused by four serotypes of DENV. Neg, negative control; P, positive control. (**D**) Nrf2 nuclear translocation (*green*) in DENV-infected human monocytes (E protein positive, *red*). (**E**) DENV-activated ARE in RAW264.7 cells and ML1 cells. (**F**) Nrf2 expression was silenced in RAW264.7 cells by using a lentiviral-based shRNA (shNrf2). Following DENV infection, ARE activity was detected. Luciferase shRNA (shLuc) was used as a negative control. (**G**) ARE activity assay in RAW264.7 cells pretreated with or without all-trans retinoic acid (ATRA) (1 μM) for 12 h and then infected with DENV. DMSO was used as the negative control. For Western blot results, the relative protein expression was determined by the ratio of the detected proteins to an internal β-actin control. Full-length blots/gels are presented in Supplementary Fig. 1. Values of all quantified data are presented as the mean ± SD of three independent experiments. *p < 0.05, **p < 0.01, and ***p < 0.001 compared to MOCK. ^#^*p* < 0.05 compared to DENV.

We created an *in vitro* model of DENV infection using murine monocytic RAW264.7 cells. After cells were infected with DENV serotype 2 PL046, electron microscopy was performed, which showed that upon viral entry, NS1 protein levels considerably increased (Fig. 1A, top). To investigate differential expressions of TFs during DENV infection, we performed high-throughput screening using the Cignal^TM^ finder pathway reporter array to determine activation levels of 45 TFs after 6 h of DENV infection. In addition to STAT1, which is a well-known downstream TF of the type I IFN response induced by viral infection^16^, the activity of Nrf2/Nrf1 increased most significantly (*p* < 0.001) after DENV infection (Fig. 1A, bottom). Because both Nrf2 and Nrf1 can bind to an antioxidant response element (ARE) motif, we further examined the nuclear translocation of Nrf2 and Nrf1 by confocal microscopy, and results showed that Nrf2 but not Nrf1 was significantly (p < 0.05) translocated into nuclei (Fig. 1B). To check whether this phenomenon is serotype-specific, RAW264.7 cells were infected with DENV serotypes 1~4, and all serotypes of DENV significantly (*p* < 0.01) induced ARE activation (Fig. 1C). Consistent with this observation, the nuclear translocation of Nrf2 also significantly (*p* < 0.05) increased in DENV-infected human primary monocytes (Fig. 1D). Furthermore, DENV induced ARE activation in RAW264.7 cells and also in ML-1 mouse liver cells (Fig. 1E). Upon knockdown of Nrf2 expression by lentiviral short hairpin (sh)RNA (Fig. 1F, left S1), DENV-induced ARE activation was significantly (*p* < 0.05) inhibited (Fig. 1F). Treating cells with ATRA, a potent inhibitor of Nrf2^17^, significantly (*p* < 0.05) decreased DENV-induced ARE activity (Fig. 1G). These results demonstrate that Nrf2 is activated during DENV infection.

### DENV induces Nrf2 activation through ER stress-PERK signaling.

**Figure 2:**
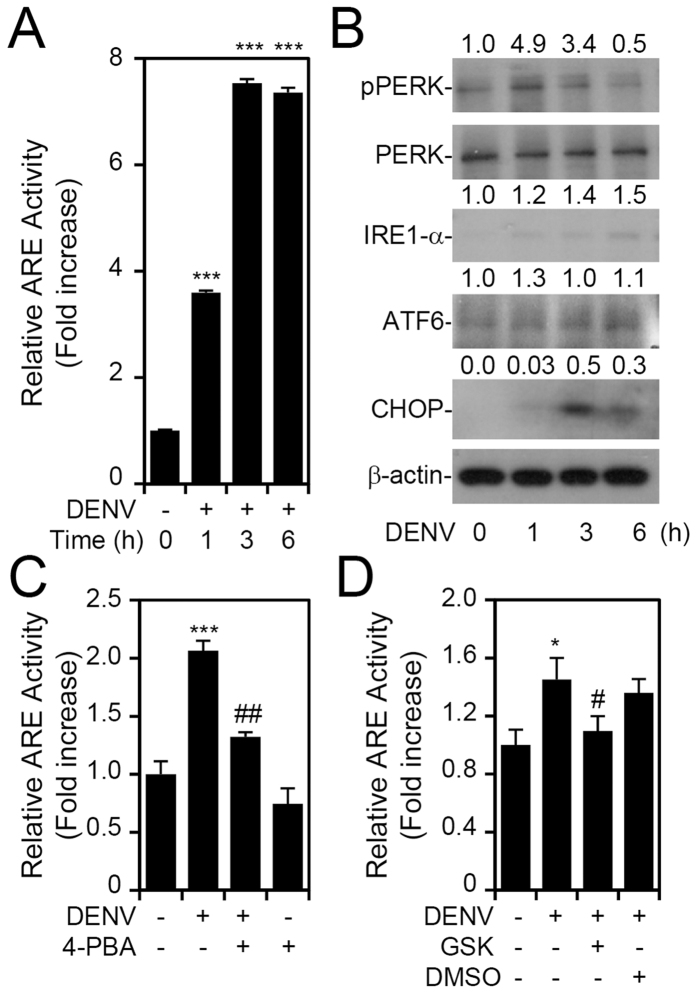
Dengue virus (DENV) infection activates nuclear factor (erythroid-derived 2)-like 2 (Nrf2) through an endoplasmic reticular (ER) stress-protein kinase RNA-like endoplasmic reticular (ER) kinase (PERK) pathway. Antioxidant response element (ARE) reporter assay (**A**) and Western blot analysis (**B**) were used to detect the time-kinetic Nrf2 activation and signaling of ER stress, respectively, in DENV2-infected RAW264.7 cells. For Western blot results, the relative protein expression was determined by the ratio of the detected proteins to an internal β-actin control. Full-length blots/gels are presented in Supplementary Fig. 2. Pharmacological inhibition of ER stress (**C**) and PERK (**D**) using 4-PBA (5 mM) and GSK2606414 (1 μM), respectively, was further evaluated for their protective effects against DENV-induced ARE activity. Values of the ARE activity assay are presented as the mean ± SD of three independent experiments. *p < 0.05, ***p < 0.001 compared to MOCK; ^#^*p* < 0.05 compared to DENV.

A previous study revealed that DENV can induce an unfolded protein response in a time-dependent manner^18^. In the early stage of DENV infection, PERK is activated and the IRE1 and ATF6 pathways are respectively triggered in mid and late stages. On the other hand, ER stress can also activate Nrf2 by IRE1α-ASK1-JNK- or PERK-mediated Nrf2 phosphorylation^19^,^20^,^21^,^22^. Considering that DENV infection can induce ER stress, which in turn can induce Nrf2, we sought to investigate the role of DENV-induced ER stress in Nrf2 activation. We first checked the time course of Nrf2 activation after DENV infection, and results revealed that ARE activity significantly (*p* < 0.001) increased after 1 h post-infection (Fig. 2A). To investigate which unfolded protein response pathway might be involved in this early stage, we profiled protein expressions of phosphorylated (p)-PERK, PERK, activated ATF6, and IRE1α by Western blotting. After DENV infection, p-PERK was significantly upregulated at an earlier time point, while levels of the chaperone protein, CHOP, increased at a later time point (Fig. 2B, S2). We then treated cells with the ER stress inhibitor, 4-PBA (Fig. 2C), which is a chemical chaperone, and the PERK inhibitor, GSK2606414 (Fig. 2D); both of which resulted in a decrease in DENV-induced ARE activation. However, pharmacologically inhibiting JNK did not alter DENV-induced ARE activation (data not shown). These results suggest that DENV infection induces ER stress, which causes PERK phosphorylation to activate Nrf2.

### Nrf2 determines CLEC5A expression by targeting the *Clec5a* gene during DENV infection

**Figure 3:**
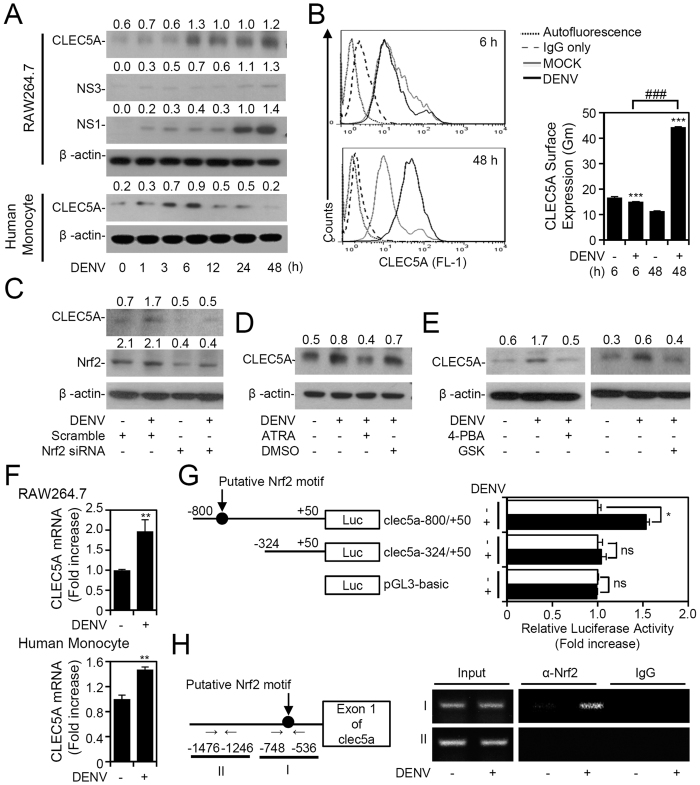
Signaling of endoplasmic reticular (ER) stress-nuclear factor (erythroid-derived 2)-like 2 (Nrf2) upregulates C-type lectin domain family 5, member A (CLEC5A) through binding to the promoter directly during dengue virus (DENV) infection. (**A**) Time-kinetic expression of CLEC5A in DENV-infected RAW264.7 cells and human monocytes. (**B**) Flow cytometric analysis of the surface expression of CLEC5A in DENV-infected RAW264.7 cells. Data are shown as the mean fluorescence intensity. Western blot analysis of CLEC5A in Nrf2 siRNA-transfected (**C**), all-trans retinoic acid (ATRA; 1 μM)-treated (**D**), and 4-PBA (5 mM)-treated, or GSK2606414 (1 μM)-treated (**E**) RAW264.7 cells with DENV infection. Scrambled siRNA and DMSO were used as negative controls. (**F**) qPCR analysis of CLEC5A mRNA expression in DENV-infected RAW264.7 cells and human monocytes. (**G**) Schematic representation of CLEC5A promoter constructs with or without a putative Nrf2 binding site (circle) used in the study. The reporter assay showed transactivation of the CLEC5A promoter after DENV infection in RAW264.7 cells. (**H**) A ChIP assay was carried out to detect a CLEC5A promoter fragment encompassing the proximal Nrf2-binding site (I) or apart from the Nrf2-binding site as a negative control (II). For Western blot results, the relative protein expression was determined as the ratio of detected proteins to an internal β-actin protein. Full-length blots/gels are presented in Supplementary Fig. 3. Values of all quantified data are presented as the mean ± SD of three independent experiments. *p < 0.05, **p < 0.01, and ***p < 0.001 compared to MOCK. ^###^*p* < 0.001.

A previous report showed that CLEC5A is one of the target genes of Nrf2^15^; however, there is no evidence to suggest a link between DENV infection and CLEC5A expression. To determine whether DENV-induced Nrf2 triggers an increase in CLEC5A expression, we found that there was a significant increase in CLEC5A levels from 6 to 48 h post DENV infection (Fig. 3A, top, S3). Consistent with results from RAW264.7 cells, infection of human primary monocytes with the DENV also caused upregulation of CLEC5A (Fig. 3A, bottom, S3). The surface expression of CLEC5A also increased following DENV infection as confirmed by flow cytometry (Fig. 3B). Inhibiting Nrf2 by either siRNA (Fig. 3C, S3) or ATRA (Fig. 3D, S3) blocked DENV-induced CLEC5A upregulation. In addition, pharmacological blocking of ER stress and PERK by GSK2606414 and 4-PBA, respectively, also caused a decrease in DENV-induced CELC5A expression (Fig. 3E, S3). These results reveal that DENV infection upregulates CLEC5A through an ER stress-PERK-Nrf2-regulated pathway.

Based on results of a genome-wide ChIP-sequencing analysis from Hirotsu *et al.*, the binding site for Nrf2 is located on the promoter of *Clec5a* from bases −781 to −325^15^. We also identified this putative Nrf2-binding motif using the online software, PROMO3. To explore whether Nrf2 can directly bind to this promoter region in *Clec5a* to drive mRNA expression during DENV infection, we performed a real-time qPCR, which showed that DENV induces *Clec5a* mRNA expression in both RAW264.7 cells and human primary monocytes (Fig. 3F). We then constructed the promoter region of *Clec5a* in a pGL3-basic reporter plasmid that contained the promoter region either from −800 to +50 bp or from −324 to +50 bp, which removes the putative binding region. The plasmid containing the promoter region showed high activation after DENV infection, while the deletion plasmid did not. Using this deletion reporter assay, we identified a potent Nrf2-responsive region in the *Clec5a* promoter at −800/−325 bp following DENV infection (Fig. 3G). In addition, an *in vivo* DNA-binding assay using ChIP showed that binding of Nrf2 to the *Clec5a* promoter was responsive to DENV infection in RAW264.7 cells (Fig. 3H). These results indicate that Nrf2 can directly bind to the promoter of *Clec5a* during DENV infection.

### Signaling of Nrf2-CLEC5A contributes to TNF-α production during DENV infection

**Figure 4:**
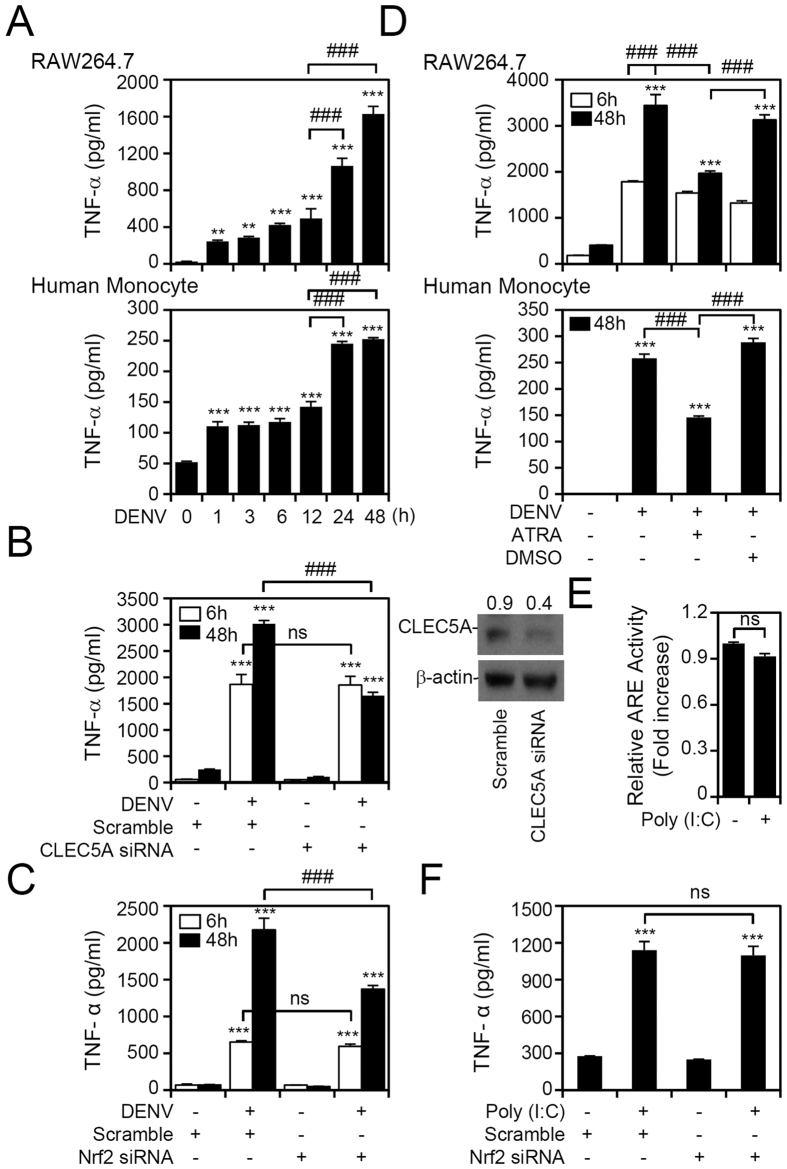
Signaling of nuclear factor (erythroid-derived 2)-like 2 (Nrf2)-C-type lectin domain family 5, member A (CLEC5A) contributes to Toll-like receptor 3 (TLR3)-independent tumor necrosis factor (TNF)-α production during dengue virus (DENV) infection. (**A**) Time-kinetic production of TNF-α in DENV-infected RAW264.7 cells and human monocytes. (**B**) Western blot analysis showing the expression of CLEC5A in CLEC5A-silenced RAW264.7 cells. Following DENV infection for 6 and 48 h, TNF-α production was measured by an ELISA. The relative protein expression was determined by the ratio of the detected proteins to an internal β-actin control. Scrambled siRNA was used as a negative control. Full-length blots/gels are presented in Supplementary Fig. 4. At 6 and 48 h post-infection, DENV induced TNF-α production in Nrf2-silenced (**C**) and all-trans retinoic acid (ATRA; 1 μM)-treated (**D**) RAW264.7 cells and human monocytes. DMSO was the negative control. (**E**) Antioxidant response element (ARE) reporter assay in poly(I:C) (50 μg/ml)-treated RAW264.7 cells for 24 h. (**F**) TNF-α production in poly(I:C) (50 μg/ml)-treated RAW264.7 cells transfected with scrambled or Nrf2 siRNA. Values of all quantified data are presented as the mean ± SD of three independent experiments. *p < 0.05, **p < 0.01, ***p < 0.001, compared to MOCK; ^###^*p* < 0.001. ns, not significant.

CLEC5A is involved in DENV-induced TNF-α production^8^. To determine whether signaling of Nrf2-CLEC5A is involved in DENV-induced TNF-α production, we probed levels of TNF-α upon DENV infection. These results show there was an apparent burst of TNF-α induction at 24 and 48 h post-infection not only in RAW264.7 cells but also in human monocytes, which suggests a possible role for Nrf2-CLEC5A signaling in TNF-α production in the late stage of DENV infection (Fig. 4A). After using siRNA to knock down CLEC5A (Fig. 4B, S4) and Nrf2 (Fig. 4C), TNF-α production was strongly attenuated at 48 h but was not affected at 6 h post DENV infection. Consistently, treatment with ATRA also reduced TNF-α production at 48 h after DENV infection in both RAW264.7 cells and human monocytes (Fig. 4D). Considering that TNF-α is upregulated in the early stages of DENV infection, a TLR3-regulated TNF-α production mechanism was proposed. However, treatment with poly(I:C), a synthetic analog of double-stranded RNA, which is an agonist of TLR3, did not alter ARE activity (Fig. 4E). Furthermore, silencing Nrf2 had no effects on poly(I:C)-induced TNF-α production (Fig. 4F). These results demonstrate that signaling of Nrf2-CLEC5A is responsible for TLR3-independent TNF-α induction during late stages of DENV infection.

### DENV NS2B3 is involved in ER stress induction, Nrf2 activation, CLEC5A upregulation, and TNF-α production

**Figure 5:**
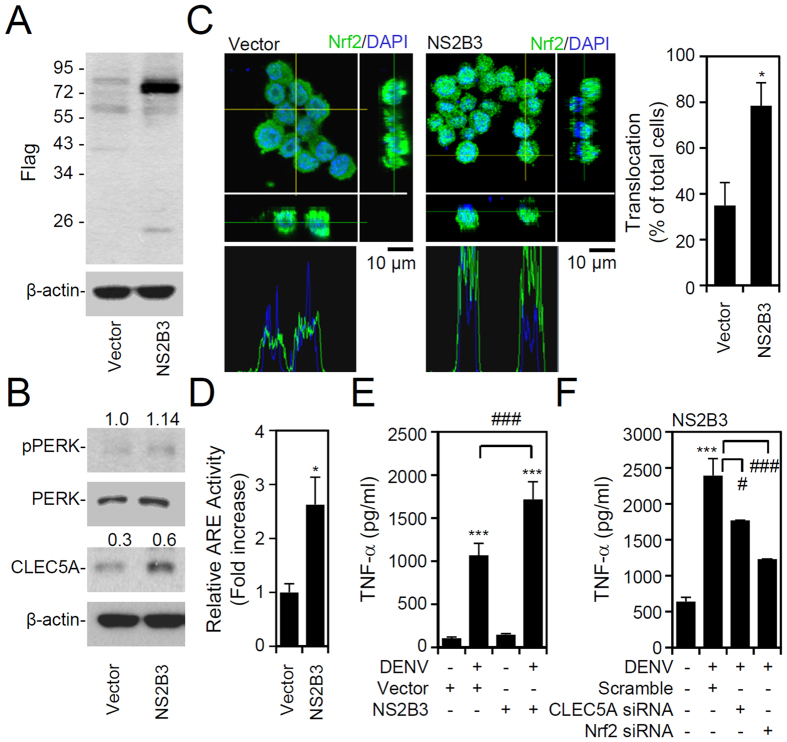
Impact of dengue virus (DENV) NS2B3 on endoplasmic reticular (ER) stress, nuclear factor (erythroid-derived 2)-like 2 (Nrf2) activation, and tumor necrosis factor (TNF)-α production. RAW264.7 cells were transfected with the pCR3.1 vector (vector) and pCR3.1-NS2B3-Flag (NS2B3). A Western blot analysis showed the expression of Flag (**A**), phosphorylated PERK Thr981, PERK, and CLEC5A (**B**). The relative protein expression was determined by the ratio of the detected proteins to an internal β-actin control. Full-length blots/gels are presented in Supplementary Fig. 5. (**C**) Meanwhile, confocal immunostaining for nuclear translocation of Nrf2 (*green*) in transfected cells was quantified and is shown as a percentage. DAPI was used as a nuclear stain (*blue*). (**D**) An antioxidant response element (ARE) activity assay was performed to illustrate Nrf2 activation induced by NS2B3. After DENV infection for 48 h, TNF-α production is shown in NS2B3-transfected RAW264.7 cells without (**E**) or with (**F**) C-type lectin domain family 5, member A (CLEC5A) and Nrf2 silencing. Values of all quantified data are presented as the mean ± SD of three independent experiments. *p < 0.05 and ***p < 0.001, compared to MOCK; ^##^*p* < 0.01 and ^###^*p* < 0.001.

Our results demonstrated that ER stress mediates Nrf2 activation during DENV infection (Fig. 2). Previous studies also identified that forced expression of NS2B3, neither NS2B nor NS3, considerably induces ER stress in a protease- and helicase-independent manner^23^. We therefore used NS2B3 overexpression (Fig. 5A, S5) to investigate the effect of NS2B3-induced ER stress on the signaling axis of Nrf2-CLEC5A-TNF-α. A further Western blot analysis showed an increase in PERK phosphorylation and upregulation of CLEC5A expression in cells overexpressing NS2B3 (Fig. 5B, S5). Nrf2 was also significantly (*p* < 0.05) translocated to nuclei of cells overexpressing NS2B3 (Fig. 5C). Furthermore, ARE activation was induced by NS2B3 overexpression (Fig. 5D). In contrast, overexpressing NS3 alone, which has no ability to induce ER stress, caused no effect on ARE activation or Nrf2 nuclear translocation (Fig. S6A–C). By overexpressing NS2B3, these cells showed increases in p-PERK and CLEC5A expressions, and Nrf2 nuclear translocation (Fig. S6D–G). In addition to inducing Nrf2 and ARE activation, NS2B3-overexpressing cells exhibited increased TNF-α production in a DENV-dependent manner (Fig. 5E). Silencing of Nrf2 or CLEC5A by siRNA in DENV-infected cells blocked NS2B3 overexpression-enhanced TNF-α production (Fig. 5F). These data suggest that DENV NS2B3 causes ER stress to activate PERK-regulated Nrf2 activation, CLEC5A expression, and TNF-α production.

### ATRA treatment suppresses CLEC5A expression and TNF-α production in a suckling mice model

**Figure 6:**
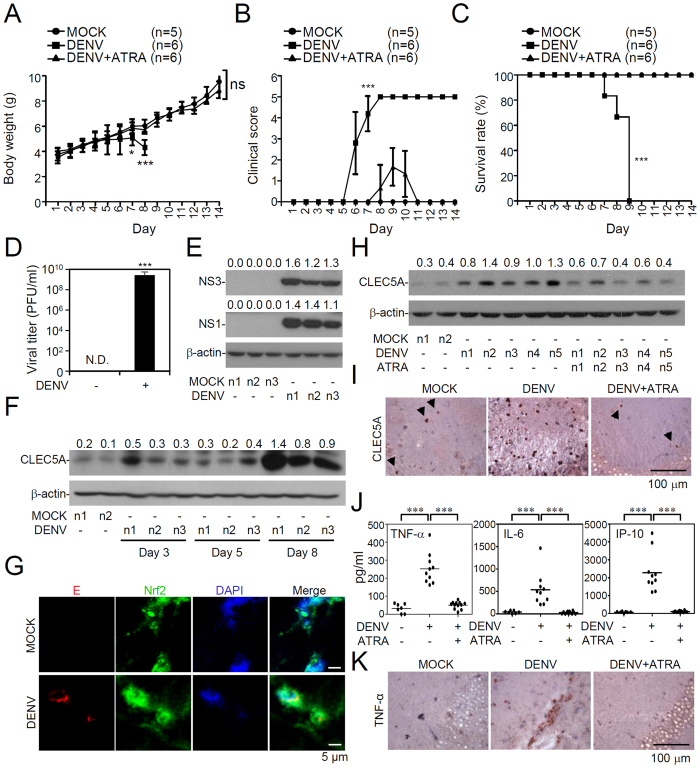
All-trans retinoic acid (ATRA) inhibits C-type lectin domain family 5, member A (CLEC5A) and tumor necrosis factor (TNF)-α expression, and increases the survival rate in suckling mice during dengue virus (DENV) infection. With or without ATRA treatment, 7-day-old ICR suckling mice were concurrently inoculated with DENV2 PL046 by intracranial and intraperitoneal injections. Time-kinetic changes in body weight (**A**), clinical scores (**B**), and survival rates (**C**). *p *<* 0.05 and ***p *<* 0.001 compared to MOCK. ns, not significant. (**D**) Plaque assay of DENV replication in the brain of ICR suckling mice 8 days post-infection (*n* = 5). Values are presented as the mean ± SD of three independent experiments. ***p < 0.001 compared to MOCK. ND, non-detected. (**E**) Meanwhile, Western blots illustrate brain expression of DENV NS1 and NS3. (**F**) Time-kinetic expression of C-type lectin domain family 5, member A (CLEC5A) in DENV-infected brains of ICR suckling mice. (**G**) Representative confocal immunostaining of DENV E protein (*red*) and Nrf2 (*green*) in the brain of DENV-infected mice 5 days post-infection. DAPI (*blue*), a nuclear stain. Following DENV infection for 8 days with or without ATRA treatment (10 mg/kg), (**H**) Western blotting showed CLEC5A expression; (**I**) IHC staining displayed CLEC5A expression; and ELISA (**J**) and IHC staining (**K**) showed cytokine production. Arrowheads indicate CLEC5A-positive cells. ***p < 0.001 and ###*p* < 0.001. For Western blot results, the relative protein expression was determined by the ratio of the detected proteins to an internal β-actin control. Full-length blots/gels are presented in Supplementary Fig. 7.

To clarify this novel mechanism both *in vitro* and *in vivo*, 7-day-old ICR suckling mice were infected with DENV through concurrent intraperitoneal and intracranial routes as established by our previous works^24^. We first monitored a time-kinetic change in body weight, clinical scores, and survival rates of infected mice with or without ATRA for 14 days post-infection. Compared to MOCK-infected mice, a significant decline in body weight (Fig. 6A) and a significant increase in clinical scores (Fig. 6B) occurred in DENV-infected mice 7 days post-infection. Survival rates of DENV-infected mice were 80% and 60% on days 7 and 8 post-infection, respectively, and all of the mice had died by day 9 post-infection (Fig. 6C). Notably, ATRA apparently blocked DENV-induced loss of body weight, increase in clinical scores, and death. In brain tissues, a high viral titer (Fig. 6D) and the presence of viral protein expression (Figs 6E and S7), including NS1 and NS3, were identified in DENV-infected mice. Importantly, CLEC5A expression in the brain was strongly induced following DENV infection (Figs 6F and S7). Furthermore, nuclear translocation of Nrf2 was found in DENV-infected cells (Fig. 6G). ATRA treatment decreased CLEC5A expression in DENV-infected mice according to a Western blot analysis (Figs 6H and S7) and IHC staining (Fig. 6I). Using an ELISA (Fig. 6J) and IHC staining (Fig. 6K), we found that ATRA treatment also reduced DENV-induced not only TNF-α but also IL-6 and IP-10 production. These results demonstrate that DENV induces CLEC5A expression and TNF-α production through Nrf2 activation *in vivo*.

## Discussion

**Figure 7:**
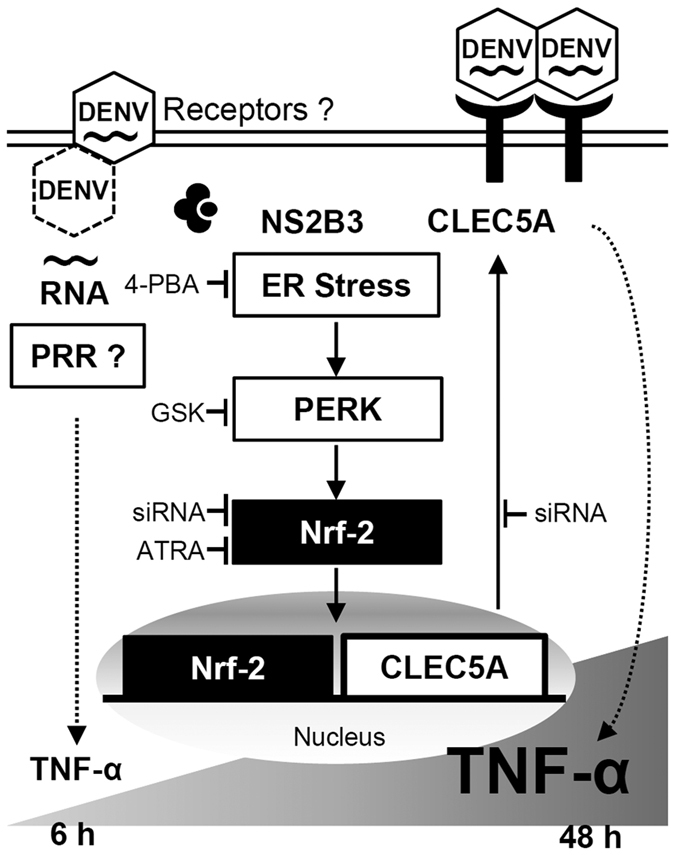
Schematic model of dengue virus (DENV)-induced nuclear factor (erythroid-derived 2)-like 2 (Nrf2)-regulated tumor necrosis factor (TNF)-α production by enhancing C-type lectin family 5, member A (CLEC5A) expression.

Herein, we identified novel regulation for late TNF-α production during DENV infection that is distinct from the conventional inflammatory signaling of pattern recognition receptors (PRRs) for early TNF-α production. As summarized in Fig. 7, DENV infection induces ER stress-PERK, at least in part through the viral protein NS2B3 that then activates Nrf2, which was identified from a screening of 45 TFs in DENV-infected cells. Activated Nrf2 is then translocated to nuclei and binds to the promoter of CLEC5A to drive its mRNA and protein expressions. Increasing surface levels of CELC5A enhance TNF-α production in the late stage of DENV infection. This finding provides a new mechanism for viral proteins contributing to pathogenic and overwhelming TNF-α production, beyond the PRR signaling-induced early physiological induction of TNF-α.

An increase in TNF-α production, which has a well-established role in promoting vascular leakage, shows a positive correlation with the severity of illness in patients with DHF and DSS^25^,^26^,^27^,^28^,^29^. Single-nucleotide polymorphisms of TNF-α at the TNF-308A allele were also identified as a risk factor for hemorrhagic manifestations in severe dengue patients^30^. Despite the controversial role of TNF-α in DENV pathology, several lines of evidence support the idea that TNF-α production contributes to hemorrhage development during DENV infection^31^,^32^,^33^. In a mouse model, percentages of TNF-α knockout mice that developed hemorrhage were highly diminished compared to wild-type mice after DENV infection^32^. Furthermore, treatment with an anti-TNF-α neutralizing antibody significantly reduced mortality associated with vascular leakage under DENV infection^33^,^34^. *In vitro* studies showed that TNF-α enhances DENV-induced apoptosis in primary vascular endothelial cells^31^,^32^. An animal model is currently established according to our recent study^24^. In this model, cytokine analysis of DENV-infected brain was carried out by cytokine array. According to the results, RANTES, CXCL-16, sTNFR1, IL-12, IFN-γ, MCP-5, IL-6, and MCP-1 were significantly increased in the infected brains. It is interesting to study whether there are different cytokine responses resulted from infections in the brain and other organs. Furthermore, in this model, DENV infection might cause significant mortality in mice. The role of virus replication and cytokine response in DENV-induced lethality need further study. All of findings suggest a pathogenic role for TNF-α in dengue-associated hemorrhagic disorders; however, regulation of TNF-α production still needs to be investigated.

It is generally suggested that viral infection triggers proinflammatory cytokines through interactions of viral pathogen-associated molecular patterns with PRRs. In addition, PRRs are involved in DENV-induced TNF-α production, and it was recently demonstrated that DENV NS1 protein promotes TNF-α production via TLR2, TLR4, and TLR6^35^,^36^. Additionally, other PRRs, including retinoic-acid-inducible gene I, melanoma differentiation-associated gene 5, and TLR3, play important roles in antiviral IFN responses to restrict DENV replication^37^. In addition to PRRs, moreover, signaling pathways of NF-κB, p38 mitogen-activated protein kinase (MAPK), and CD137 were also suggested to participate in DENV-induced TNF-α production^14^,^38^. Through the proinflammatory DENV receptor, CLEC5A, DENV infection may cause PRR-independent TNF-α production to facilitate hemorrhagic disorders^8^. In this study, CLEC5A silence only decreased the late stage of TNF-α production, indicating a CLEC5A-regulated manner. Moreover, through a ploy(I:C) treatment as mimic with TLR3 signaling, there is no effect on TNF-α production in cells with Nrf2 and/or CLEC5A silence. These results suggest several regulatory pathways of TNF-α production could be activated by DENV infection and regulation of CLEC5A may be vital for DENV-induced TNF-α overproduction.

CLEC5A was also demonstrated to be involved in DENV-induced inflammatory signaling, inflammasome activation, and lethality^8^,^39^,^40^. However, regulation of CLEC5A is still largely unknown. The activated TF, PU.1, can bind to the promoter of *Clec5a* in differentiated myeloid lineage cells^41^. Importantly, a recent study found a global regulation mechanism of Nrf2-targeted genes with *Clec5a* being one of its targets^15^. Nrf2 exerts a protective role against oxidative injury via binding to the ARE to drive expressions of antioxidant and cytoprotective genes^42^. Under different stimuli, including oxidative and ER stress and other chemical inducers, Nrf2 is rapidly activated^22^,^42^,^43^. Activated Nrf2 can then be translocated to nuclei where it binds to ARE motifs on different gene promoters^42^. Additionally, increasing numbers of studies have revealed that Nrf2 is activated during several viral infections^44^. For the first time, we showed a possible pathway that through ER stress, at least PERK- but not IRE1α-ASK1-JNK-regulated pathway, DENV activates Nrf2 to facilitate CLEC5A expression followed by induction of TNF-α production.

Herein, we found that the DENV viral protein, NS2B3, promoted CLEC5A expression through ER stress-induced Nrf2 activation. NS2B3 can be activated by DENV infection to facilitate viral protein cleavage as well as immune escape from type I IFN response^45^,^46^. Basically, NS2B3 can abrogate IRF3 phosphorylation, but has no impact on NF-κB activation during DENV infection^47^. For NS2B3-induced inflammation, our previous work^14^ and others^48^ showed NS2B3 causes NF-κB-regulated TNF-α production. Previous works also demonstrated that NS2B3 may bind to inhibitors of κB to activate NF-κB^49^. These findings suggest NS2B3 may also act as pro-inflammatory inducer after DENV infection.

Recently, Olagnier *et al.* identified that DENV infection induces reactive oxygen species (ROS) required for the Nrf2-mediated antioxidant response as well as inflammatory and antiviral responses in monocyte-derived dendritic cells^50^. Consistent with their results, we also observed that DENV activated Nrf2 at 6 h, but we could not detect ROS generation by RAW264.7 cells (data not shown). They also showed that knockdown of Nrf2 enhances some antiviral and inflammatory genes through upregulating ROS. However, augmentation of antiviral genes was not reflected in levels of viral RNA. Only in respiratory syncytial virus and influenza A virus infection models does Nrf2 confer similar effects on viral replication^51^,^52^. However, there was no effect of Nrf2 activation on viral replication in the hepatitis B virus or herpes simplex virus^53^,^54^. A study of Nrf2 in hepatitis C virus infection demonstrated that Nrf2 induced a cell-survival pathway, but researchers did not clarify how this affects viral replication^55^. A similar study revealed that Nrf2 is hijacked by the Marburg virus via targeting of the Kelch domain of Kelch-like ECH-associated protein 1 to ensure survival of infected cells. However, its role in viral replication needs further investigation^56^. Although CLEC5A was not directly involved in DENV replication, it will take more experiments to clarify the roles of Nrf2 in DENV infection, not only in terms of TNF-α production but also for cell survival, which may be key for DENV replication^57^.

Several studies used large-scale screening to identify host factors involved in different cell fates including cell cycle regulation, cell death, ROS generation, lipid metabolism, and inflammation, and these were shown to be affected by DENV infection^58^,^59^,^60^. Increasing numbers of host factors are reported to be involved in different aspects of DENV pathogenesis, including entry and signaling, replication, assembly and egress, as well as immune invasion and pathogenesis^61^. Targeting host factors not only influences viral replication or infection but also affects the host response, which further contributes to viral pathogenesis and innate immunity^62^,^63^. Hence, multiple drugs which manipulate these host factors may serve as potential candidates for the future development of therapeutic agents^63^. In this study, we demonstrated that DENV activates Nrf2 through ER stress-PERK to enhance TNF-α production through transcriptionally upregulating CLEC5A in mononuclear phagocytes. This study provides new insights into DENV-induced hemorrhagic disorders by targeting not only CLEC5A and TNF-α but also Nrf2.

## Methods

### Ethics statement

Animal studies of this project were performed according to the rules of the *Animal Protection Act* of Taiwan, and all protocols according to guidelines established by the Ministry of Science and Technology, Taiwan were approved by the Laboratory Animal Care and Use Committee of National Cheng Kung University (IACUC #104062).

### Antibodies (Abs) and reagents

Reagents and Abs used were GSK2606414 (Tocris Bioscience. Bristol, UK); 4-phenylbutyric acid (4-PBA) (Calbiochem, San Diego, CA); polybrene; all-trans retinoic acid (ATRA), 4,6-diamidino-2-phenylindole (DAPI); dimethyl sulfoxide (DMSO), mouse monoclonal (m)Ab specific for β-actin (Sigma-Aldrich, ST. Louis, MO); Alexa Fluor 488-conjugated goat anti-rabbit and Alexa Fluor 594-conjugated goat anti-mouse; Abs against CLEC5A, DENV NS1, NS3, and E proteins (GeneTex, Irvine, CA); Abs against Flag and IRE1α (Cell Signaling Technology, Danvers, MA); Abs against Nrf2, phosphorylated (p)-PERK at Thr981, PERK, activated ATF6, and CHOP (Santa Cruz Biotechnology, Santa Cruz, CA); and an Ab against TNF-α (Abcam, Cambridge, MA). All drug treatments were assessed for cytotoxic effects using cytotoxicity assays prior to the experiments. Non-cytotoxic dosages were used in this study.

### Cell culture and virus culture

RAW264.7 mouse macrophages were purchased from American Type Culture Collection (ATCC #TIB-71, Manassas, VA) and were grown on plastic in Dulbecco’s modified Eagle’s medium (DMEM) with 10% fetal bovine serum (FBS) (Sigma-Aldrich), 100 units of penicillin, and 100 μg/ml of streptomycin at 37 °C under 5% CO_2_. Cells were used after 3 to 5 passages. Baby hamster kidney (BHK) cells and C6/36 cells were cultured in DMEM (Invitrogen Life Technologies, Gaithersburg, MD) containing FBS. Monocyte-enriched peripheral blood mononuclear cells (PBMCs) were isolated from healthy volunteers by density-gradient centrifugation using Ficoll-Paque Plus (GE Healthcare, Piscataway, NJ), washed twice with red blood cell lysis buffer (eBioscience, San Diego, CA), resuspended in RPMI 1640 medium supplemented with 10% heat-inactivated FBS, and maintained at 37 °C in an atmosphere containing 5% CO_2_ while allowing adherence onto uncoated polystyrene flasks for 90 min for monocyte enrichment. Non-adherent cells were gently removed by washing, after which adherent cells were collected to perform the DENV infection experiment. The protocols and procedures were approved by the institutional review board of National Cheng Kung University Hospital (no. A-ER-102-123). Four serotypes of DENV (DENV1 8700828, DENV2 PL046, DENV3 8700829A, and DENV4 59201818) were maintained in C6/36 cells. Monolayers of C6/36 cells were incubated with DENV at a multiplicity of infection (MOI) of 0.01 and incubated at 28 °C in 5% CO_2_ for 5 days. The virus supernatant was further filtered with a 0.22-μm filter, and then stored at −80 °C until use. Virus titers were determined with a plaque assay using the BHK cell line.

### DENV infection

RAW264.7 cells were seeded at a density of 10^5^ cells per well in 6-well plates overnight, followed by the addition of DMEM with DENV (at an MOI of 50; DENV1 8700828, DENV2 PL046, DENV3 8700829A, and DENV4 59201818). Human monocytes were resuspended at a concentration of 5 × 10^5^ cells/ml in RPMI with DENV (at an MOI of 10; DENV2 PL046). Cells were incubated for 90 min at 37 °C. Then, cells were washed once with medium, and incubated at 37 °C with 5% CO2. Viral supernatants were checked using plaque assays.

### Plaque assay

BHK-21 cells were plated into 12-well plates (2 × 10^5^ cells/well) and cultured in DMEM under CO_2_-enriched conditions. After adsorption with a serially diluted virus solution for 1 h, the solution was replaced with fresh DMEM containing 2% FBS and 0.5% methyl cellulose (Sigma-Aldrich). Five days post-infection, the medium was removed, and cells were fixed and stained with a crystal violet solution containing of 1% crystal violet, 0.64% NaCl, and 2% formalin.

### Plasmid overexpression

cDNA fragments of DENV2 PL046 (GenBank accession number KJ734727.1) encoding the individual viral proteins were subcloned into Flag-tagged pCR3.1 (Invitrogen) to generate pCR3.1-NS2B3 and pCR3.1-NS3 according to the previous protocols^23^. Transient transfection was performed using TurboFect Cell Transfection Reagent (Thermos, Thermo Scientific, Waltham, MA) according to the manufacturer’s instructions for optimization and usage.

### Immunostaining

To detect expressions of the Nrf1, Nrf2, and DENV2 E proteins, cells were fixed, stained, and analyzed as described elsewhere^64^. Cells were stained with anti-Nrf1, Nrf2, and DENV E Abs, and then with Alexa 488-conjugated goat anti-rabbit immunoglobulin G (IgG) and Alexa 594-conjugated goat anti-mouse IgG secondary Abs. DAPI (5 μg/ml) was used for nuclear staining. Cells were visualized under a fluorescent microscope (BX51; Olympus, Tokyo, Japan) or a laser-scanning confocal microscope (SPII; Leica Mikrosysteme Vertrieb, Wetzlar, Germany). Three-dimensional (3D) images from a series of confocal images, along with the z-axis of cells and analysis of z-stacks, were reconstructed using Leica Confocal Software (Leica Mikrosysteme Vertrieb). For the flow cytometric analysis, cells were stained with anti-CLEC5A and then incubated with a mixture of Alexa Fluor 488-conjugated secondary Abs. Cells were analyzed using flow cytometry (FACSCalibur; BD Biosciences, San Jose CA) with excitation set at 488 nm; emission was detected with the FL-1 channel (515~545 nm). Samples were analyzed using CellQuest Pro 4.0.2 software (BD Biosciences).

### Western blotting

Harvested cells were lysed in buffer containing 1% Triton X-100, 50 mM Tris (pH 7.5), 10 mM EDTA, 0.02% NaN3, and a protease inhibitor cocktail (Roche Boehringer Mannheim Diagnostics, Mannheim, Germany). After a freeze-thaw cycle, cell lysates were centrifuged at 10^4^ × *g* at 4 °C for 20 min. Lysates were boiled in sample buffer for 5 min. Proteins were then subjected to sodium dodecylsulfate polyacrylamide gel electrophoresis (SDS-PAGE) and transferred to polyvinylidene difluoride (PVDF) membranes (Millipore, Billerica, MA, USA) using a semi-dry electroblotting system. After blocking with 5% skim milk in phosphate-buffered saline (PBS), membranes were incubated overnight with a 1:1,000 dilution of primary Abs at 4 °C. Membranes were then washed with 0.05% PBS-Tween 20 and incubated with a 1:5000 dilution of a horseradish peroxidase (HRP)-conjugated secondary Ab at room temperature for 1 h. After washing, membranes were soaked in an enhanced chemiluminescent (ECL) solution (PerkinElmer Life and Analytical Sciences, Boston, MA) for 1 min and exposed to x-ray film (BioMax; Eastman Kodak, Rochester, NY). The relative signal intensity was quantified using ImageJ software (vers. 1.41o; W. Rasband, National Institutes of Health, Bethesda, MD). Changes in the ratio of proteins compared to the normalized value of untreated cells (indicated protein/β-actin) were also determined. One set of representative data obtained from three independent experiments is shown, and data are shown as the mean ± standard deviation (SD) of three independent experiments.

### Cignal^TM^ finder pathway reporter assay

Cignal™ 45-pathway reporter arrays were used to simultaneously assess 45 different signaling pathways in RAW264.7 cells. Cells were seeded into wells of the Cignal™ Finder 96-well plates (SABiosciences, Frederick, MD) to introduce pathway reporters into cells via reverse transfection according to the manufacturer’s protocol. Following transfection, cells were infected with DENV2 (PL046). Cells were then lysed, and luciferase expression was determined as described above. Results of the reporter assay are expressed as firefly luciferase activity normalized to Renilla luciferase activity and then further normalized to the quantity of mock-infected cells.

### Antioxidant response element (ARE) reporter assay

RAW264.7 cells were transfected using Turbofect with DNA plasmid constructs expressing ARE-inducible firefly luciferase, a negative (Neg) control plasmid that contained no ARE motif and a positive (Pos) control plasmid that constitutively expressed Renilla luciferase (SABiosciences) for 24 h. Cells were then incubated using the required treatments. At the end of incubation, cells were lysed, and the firefly and Renilla luciferase activities were determined by measuring the luminescence. Results of the ARE reporter assay are expressed as firefly luciferase activity normalized to Renilla luciferase activity and then further normalized to the quantity of mock-infected cells.

### Luciferase reporter assay

The longest construct (CLEC5A-800/+50) comprised sequences −800 to +50 cloned into the pGL3-basic luciferase vector, and deletion constructs were obtained by removing specific regions from the 5′ end of the promoter; that is to position −324 (CLEC5A-324/+50). The 5′ flanking regions of the CLEC5A gene were obtained by a PCR with the genomic DNA of RAW264.7 cells, and then these were individually cloned into a pGL3 basic vector. Primers for the PCR of the genomic DNA were as follows: CLEC5A-800/+50-forward primer 5′-NheI-GCTAGCAATAAAAGCCTAAAAACCAGAT-3′, and CLEC5A-800/+50-reverse primer 5′-XhoI-CTCGAGGTAATGTCCTTGTTCACAGC-3′; and CLEC5A-324/+50-forward primer 5′-NheI-GCTAGCGGACAGATTTGAGAGGTGC-3′, and CLEC5A-324/+50-reverse primer 5′-XhoI -CTCGAGGTAATGTCCTTGTTCACAGC-3′. For the reporter assay, cells were transfected with the reporters and expression vectors as indicated using Turbofect (Thermos). Lysates of transfected cells were harvested following the manufacturer’s instructions for the luciferase assay.

### Reverse-transcription (RT)-PCR and quantitative (q)PCR

Total RNA was extracted using the TRIZol (Invitrogen, Carlsbad, CA) RNA extraction reagent. The synthesis of complementary (c)DNA was completed with an RT reaction using a PrimeScript^TM^ RT reagent kit (TAKARA, Tokyo, Japan). A qPCR was conducted using KAPA SYBR FAST qPCR Master Mix (Life Technologies and Kapa Biosystems, Woburn, MA). The PCR was performed using a StepOnePlus^TM^ real-time PCR system (Applied Biosystems, Foster City, CA) with the following pairs of specific primers: primer sequences for *CLEC5A* (forward): 5′-GTT TCACCACCACCAGGAGC-3′ and *CLEC5A* (reverse): 5′-GGCATTCTTCTCACAGATCC-3′; *Clec5a* (forward): 5′-CTTCAGGACATAGCTGGTATTG-3′ and *Clec5a* (reverse): 5′-CGAAGTTCTGGTCCTGATTG-3′; *ACTB* (forward): 5′-AGAGCTACGAGCTGCCTGAC-3′ and *ACTB* (reverse): 5′-AGCACTGTGTTGGCGTACAG-3′; and *Actb* (forward): 5′-ACTGCCGCATCCTCTTCCTC-3′ and *Actb* (reverse): 5′-TGCCACAGGATTCCATACCC-3′.

### Chromatin immunoprecipitation (ChIP) assay

Briefly, RAW264.7 cells were treated with 1% formaldehyde for 15 min. Then cross-linked chromatin was prepared and sonicated until an average size of 500 base pairs was obtained. The DNA fragments were immunoprecipitated with specific Abs recognizing Nrf2 or control rabbit immunoglobulin G (IgG) at 4 °C for 12~16 h. After reversal of the crosslinking between proteins and genomic DNA, the precipitated DNA was amplified by a PCR with primers related to the specific regions on the genomic loci of target genes. The primers included CLEC5A-I (forward), 5′-GCTGCATAGTTTCAGAGTGTGG-3′ and CLEC5A-I (reverse), 5′-GACTCCTCGATGTTCTCTTTGAC-3′, or CLEC5A -II (forward), 5′-GCAGAGGGGAGTCATTCTAGTTA-3′ and CLEC5A -II (reverse), 5′-GCACATCTGTCATGGCTACTG-3′.

### TNF-α expression

After treatment, we used a commercial enzyme-linked immunosorbent assay (ELISA) kit (R&D, Minneapolis, MN) to detect the concentration of TNF-α, IL-6, and IP-10 in cell-conditioned culture medium and brain lysates of mice, according to the manufacturer’s instructions.

### RNA interference (RNAi)

Protein was downregulated using lentiviral expression of short hairpin (sh)RNA targeting mouse Nrf2 (TRCN0000012130 containing the following respective shRNA target sequences: 5′-GCTCGCATTGATCCGAGATAT-3′), and a negative control construct (luciferase shRNA, shLuc). The shRNA clones were obtained from the National RNAi Core Facility, Institute of Molecular Biology/Genomic Research Center, Academia Sinica, Taipei, Taiwan. In brief, RAW264.7 cells were transduced with a lentivirus, with an appropriate MOI, in complete growth medium supplemented with polybrene (Sigma-Aldrich). After transduction for 24 h and puromycin (Calbiochem) selection for 6 days, protein expression was monitored using a Western blot analysis. Nrf2 and CLEC5A expressions were silenced using commercialized small interfering (si)RNA (Nrf2-MSS207018 containing the following respective siRNA target sequences: 5′-GCAUGUUACGUGAUGAGGGAAA-3′and 5′-UUUCCAUCCUCAUCACGUAACAUGC-3′ for mouse Nrf2 silencing; and CLEC5A-HSS119041 containing the following respective siRNA target sequences: 59-AAUAAGCCCAGAGAUGAUCAUGUGC-39 and 59-GCACAUGAUCAUCUCUGGGCUUAUU-39 for mouse CLEC5A silencing) (Invitrogen). Transfection was performed by electroporation using a pipette-type microporator (Microporator system; Digital Bio Technology, Suwon, Korea). After transfection, RAW264.7 cells were incubated for 18 h in Dulbecco’s modified Eagle medium (DMEM) at 37 °C before infection. A nonspecific scrambled siRNA kit (Stealth^TM^ RNAi Negative Control Duplexes, 12935-100; Invitrogen) was the negative control.

**Animals.** Breeder mice of the ICR strain were purchased from the Animal Center at National Cheng Kung University. The mice were maintained and manipulated according to the animal experiment guidelines of the Ministry of Science and Technology. Seven-day-old suckling mice were inoculated intracerebrally with 2.5 × 10^5^ plaque-forming units (PFU) and intraperitoneally with 7.5 × 10^5^ of DENV2 (PL046), which was combined with or without all-trans retinoic acid (ATRA; 10 mg/kg) treatment. On day 3, 5, or 8 post-infection (p.i.), brain tissue was harvested. Brain tissue was sonicated followed by centrifugation at 2000 rpm. The lysate was subjected to a Western blot analysis, and the supernatant was subjected to a plaque assay and an ELISA. For immunohistochemical (IHC) staining, the whole brain was fixed using 4% paraformaldehyde and embedded in paraffin.

### IHC staining

Whole brains were embedded in paraffin, coronally sectioned (7 μm thick), and deparaffinized using a xylene and alcohol gradient. Sections were incubated with 3% H_2_O_2_ in methanol for 10 min to inhibit endogenous peroxidase activity and then autoclaved with 0.01 M citrate buffer for 5 min. Furthermore, each section was blocked using 5% bovine serum albumin (BSA) in phosphate-buffered saline (PBS) for 15 min. The primary and secondary Abs were adequately diluted in Ab diluents (Dako, Carpenteria, CA). Cells were detected with the indicated primary Abs overnight at 4 °C, followed by HRP-conjugated goat anti-rabbit Abs at room temperature for 1 h (Jackson Immunoresearch Laboratories, West Grove, PA). After washing with PBS, sections were incubated with streptavidin (Dako) at room temperature for 15 min. Brain sections were developed using the AEC substrate kit (Vector Laboratories, Burlingame, CA) and counterstained with hematoxylin followed by a light microscopic analysis (Olympus, Tokyo, Japan).

For immunofluorescent staining, retrieved sections were incubated with 1 M NH_4_Cl for 1 h before blocking with 5% BSA. Sections were then incubated with primary Abs at 4 °C overnight, washed, incubated for 1 h with labeled isotype-specific secondary Abs, washed, and counterstained with DAPI. Cells were visualized under a fluorescent microscope (BX51; Olympus) or a laser-scanning confocal microscope (SPII; Leica Mikrosysteme Vertrieb).

### Transmission electron microscopic (TEM) analysis

Cells were fixed with 1.2% glutaldehyde in PBS. After several washes, cells were osmicated in 1% osmium tetraoxide (Electron Microscopy Science, Washington, PA) for 1 h and then dehydrated in a graded series of ethanol, cleared in propylene oxide, and flat-embedded in EPON plastic [30% (w/w) dodecenyl succinic anhydride, 51% (w/w) EM bed-812, 18% (w/w) dadic methyl anhydride, 1% (w/w) 2,4,6-tri(dimethylaminomethyl) phenol] (Electron Microscopy Science, Hatfield, PA). Thin sections (80 nm) were examined using an electron microscope (JEOL JEM-1200EX, Jeol Ltd., Tokyo, Japan) at 75 kV.

### Statistical analysis

Data obtained from three independent experiments are presented as the mean ± standard deviation (SD). Statistical analyses of data were performed using Prism vers. 5 (GraphPad Software, San Diego, CA). Two sets of the data were analyzed by an unpaired Student’s *t*-test. Three or more sets of data were analyzed by a one-way analysis of variance (ANOVA) with Tukey’s multiple comparison post test. Statistical significance was set at *p* < 0.05.

## Additional Information

**How to cite this article**: Cheng, Y.-L. *et al.* Activation of Nrf2 by the dengue virus causes an increase in CLEC5A, which enhances TNF-α production by mononuclear phagocytes. *Sci. Rep.* **6**, 32000; doi: 10.1038/srep32000 (2016).

## Supplementary Material

Supplementary Information
