# Supplementary material for: Activation of Nrf2 by the dengue virus causes an increase in CLEC5A, which enhances TNF-α production by mononuclear phagocytes
Source: Sci Rep. 2016 Aug 26;6:32000. doi: 10.1038/srep32000 (PMC4999957; doi:10.1038/srep32000)

Activation of Nrf2 by the dengue virus causes an increase in CLEC5A, which enhances TNF-α production by mononuclear phagocytes

Yi-Lin Cheng[1,2], Yee-Shin Lin[1,2,3], Chia-Ling Chen[4], Tsung-Ting Tsai[5], Cheng-Chieh Tsai[6], Yan-Wei Wu[1,2], Yi-Dan Ou[3], Yu-Yi Chu[7], Ju-Ming Wang[1,2,7], Chia-Yi Yu[3], and Chiou-Feng Lin[2,5,8]*

[1]Institute of Basic Medical Sciences, College of Medicine, National Cheng Kung University, Tainan 701, Taiwan; [2]Center of Infectious Diseases and Signaling Research, National Cheng Kung University, Tainan 701, Taiwan; [3]Department of Microbiology and Immunology, College of Medicine, National Cheng Kung University, Tainan 701, Taiwan; [4]Translational Research Center, Taipei Medical University, Taipei 110, Taiwan; [5]Department of Microbiology and Immunology, College of Medicine, Taipei Medical University, Taipei 110, Taiwan; [6]Department of Nursing, Chung Hwa University of Medical Technology, Tainan 717, Taiwan; [7]Institute of Bioinformatics and Biosignal Transduction, National Cheng Kung University, Tainan 701, Taiwan; [8]Graduate Institute of Medical Sciences, College of Medicine, Taipei Medical University, Taipei 110, Taiwan

Supplemental figure legends

Figure S1 Protein assay for Figure 1.

Figure S2 Protein assay for Figure 2.

Figure S3 Protein assay for Figure 3.

Figure S4 Protein assay for Figure 4.

Figure S5 Protein assay for Figure 5.

Figure S6 Impact of DENV NS2B3, NS3 or GFP-Flag on ER stress, Nrf2 activation and CLEC5A expression. RAW264.7 cells were transfected with pCR3.1 vector (vector), pCR3.1-NS2B3-Flag (NS2B3), or pCR3.1-NS3-Flag (NS3). (**A**) Western blot analysis showed the expression of Flag. (**B**) ARE activity assay was performed to illustrate Nrf2 activation. (**C**) Confocal immunostaining was used to detect nuclear translocation of Nrf2 (*green*) in transfected cells. DAPI was used as a nuclear stain (*blue*). RAW264.7 cells were transfected with GFP-Flag or NS2B3-Flag. Western blot analysis showed the expression of Flag (**D**), phosphorylated PERK Thr981, PERK, and CLEC5A (**E**). The relative protein expression was determined by the ratio of the detected proteins to an internal β-actin control. (**F**) Flow cytometric analysis of the surface expression of CLEC5A in NS2B3-transfected RAW264.7 cells. The data are shown as the mean fluorescent intensity. (**G**) Confocal immunostaining was used to detect nuclear translocation of Nrf2 (*green*) in transfected cells. DAPI was used for nuclear staining (*blue*). For all quantified data, values are presented as the mean ± SD of three independent experiments. **P* < 0.05 and ***P* < 0.01, compared with vector or GFP-Flag. ns, not significant.

Figure S7 Protein assay for Figure 6.

Cheng *et al.* Supplemental Figure 1

For Figure 1A

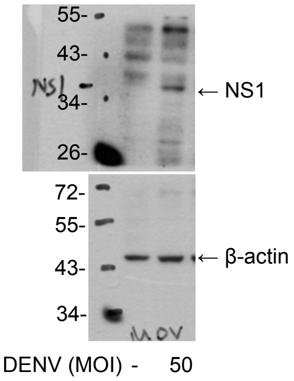

For Figure 1B

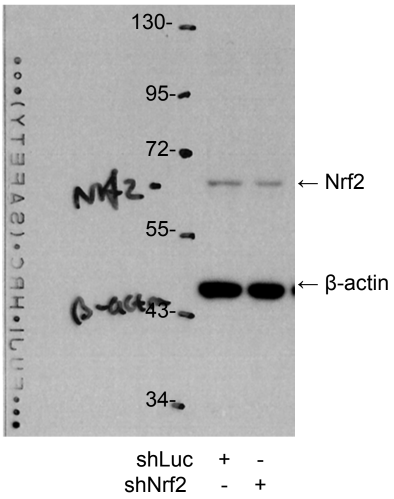

Cheng *et al.* Supplemental Figure 2

For Figure 2B

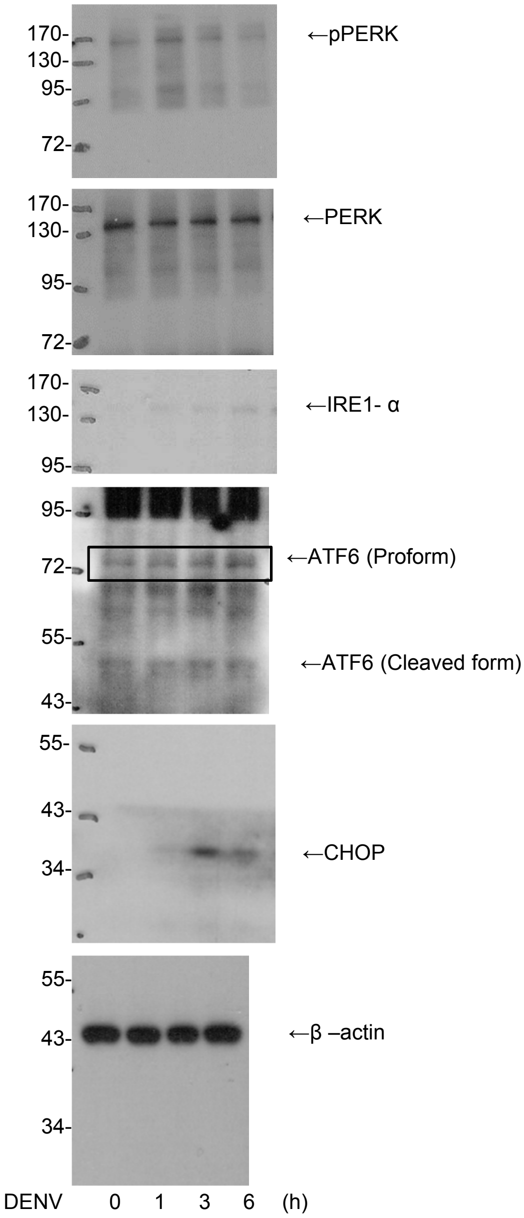

Cheng *et al.* Supplemental Figure 3

For Figure 3A

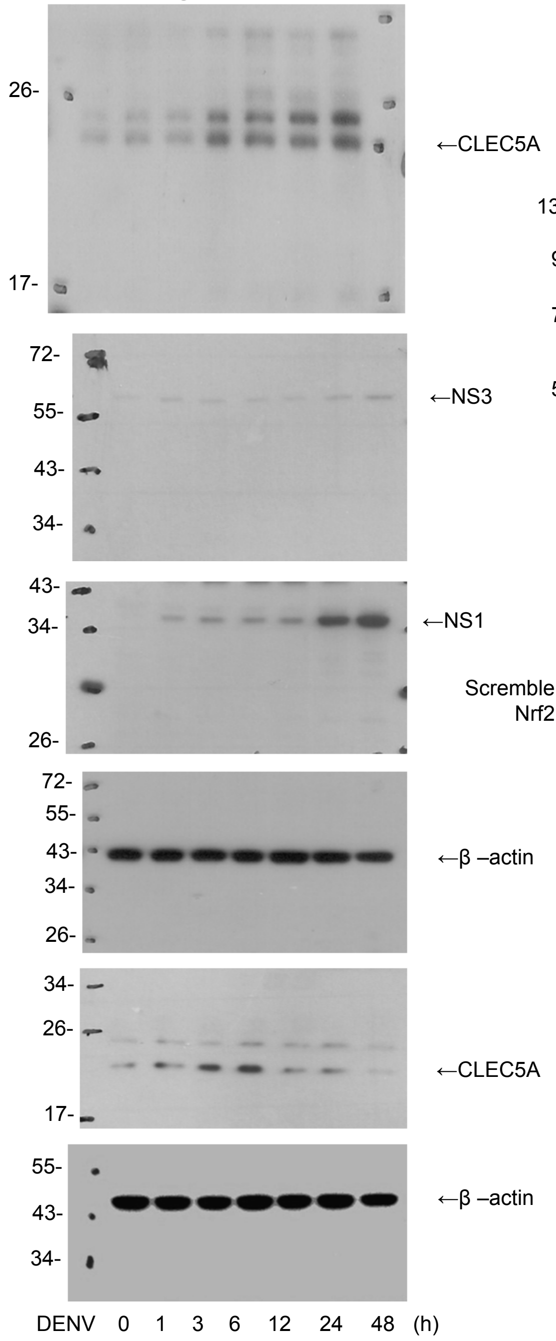

For Figure 3C

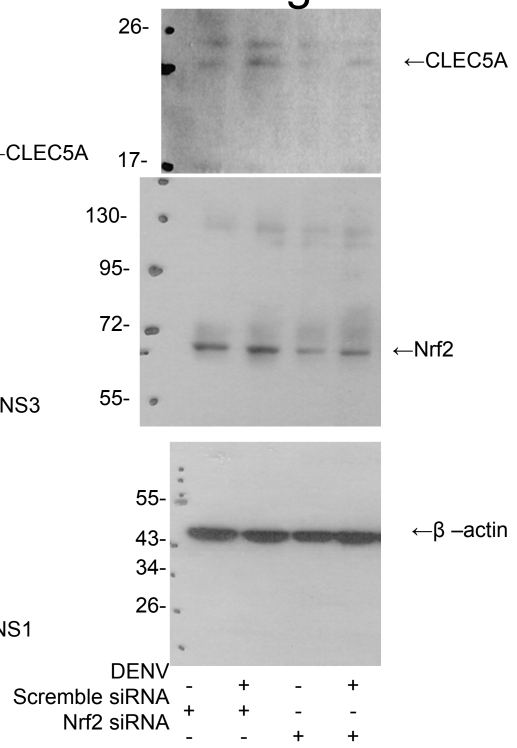

For Figure 3D

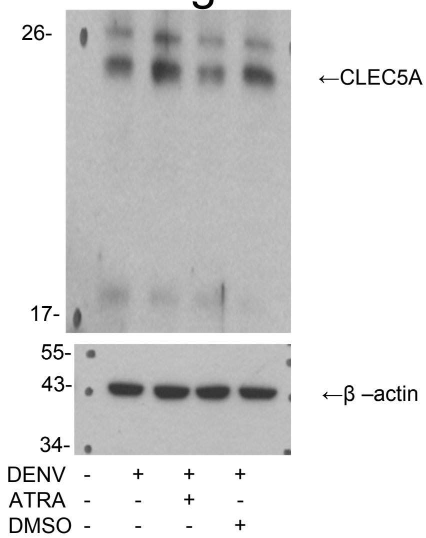

For Figure 3E

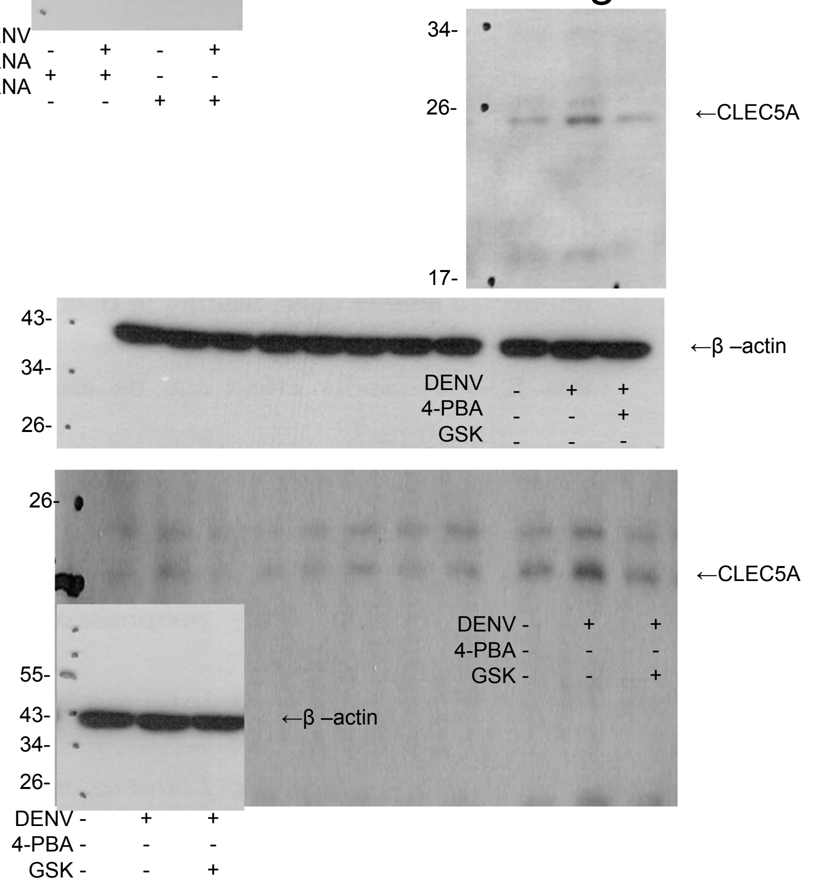

Cheng *et al.* Supplemental Figure 4

For Figure 4B

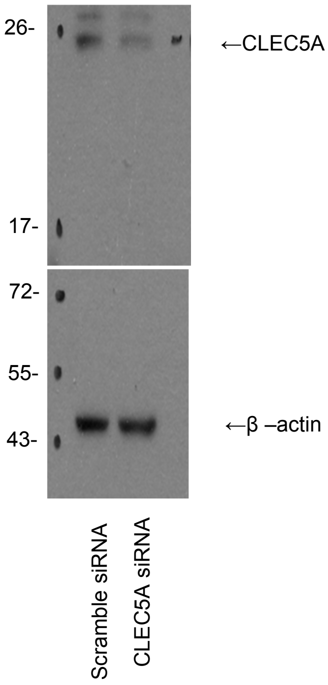

Cheng *et al.* Supplemental Figure 5

For Figure 5A

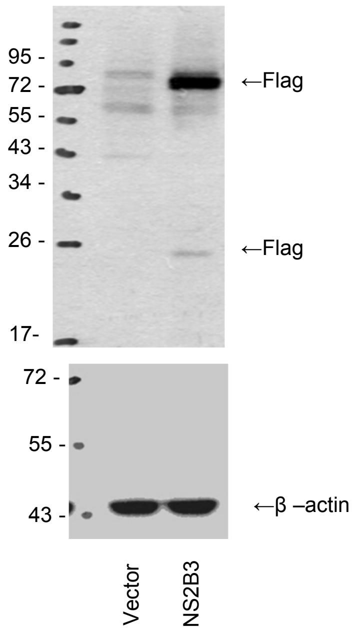

For Figure 5B

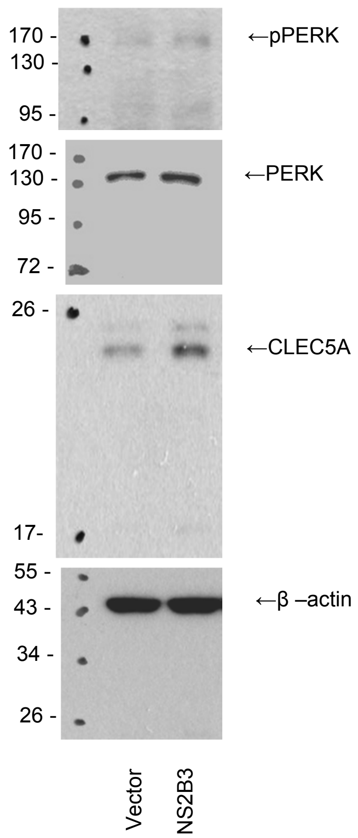

Cheng *et al.* Supplemental Figure 6

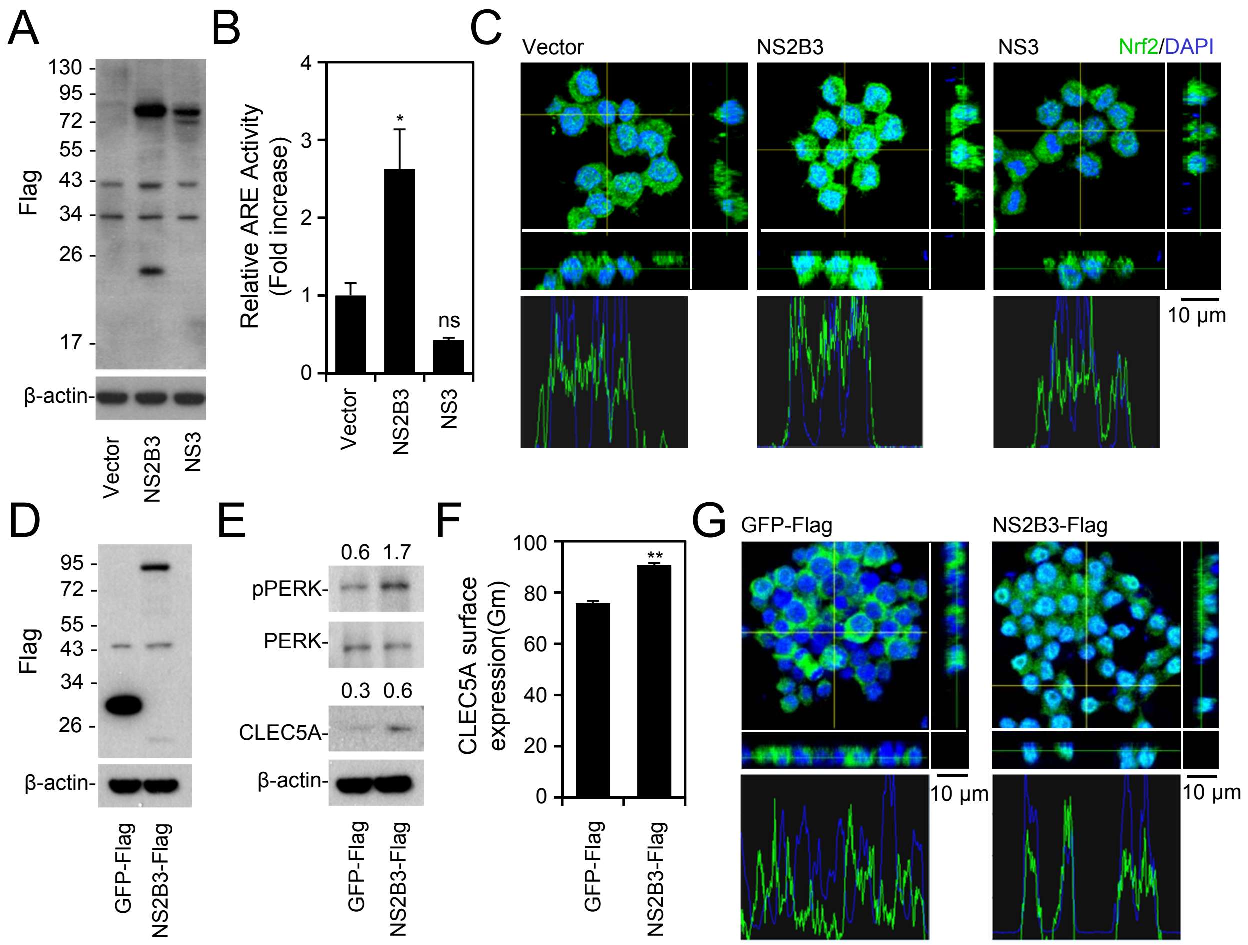

Cheng *et al.* Supplemental Figure 7

For Figure 6B

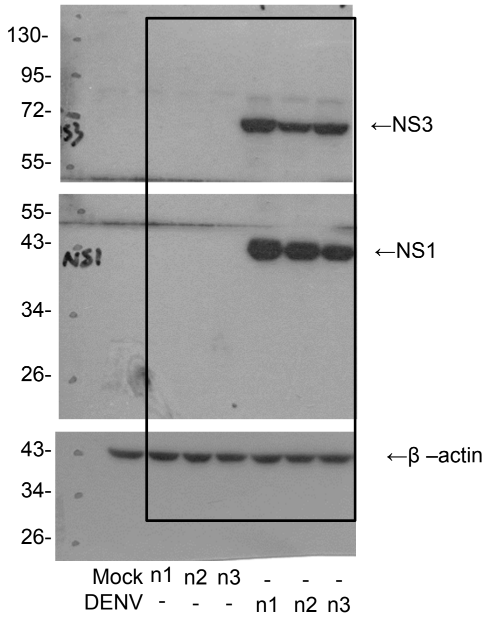

For Figure 6C

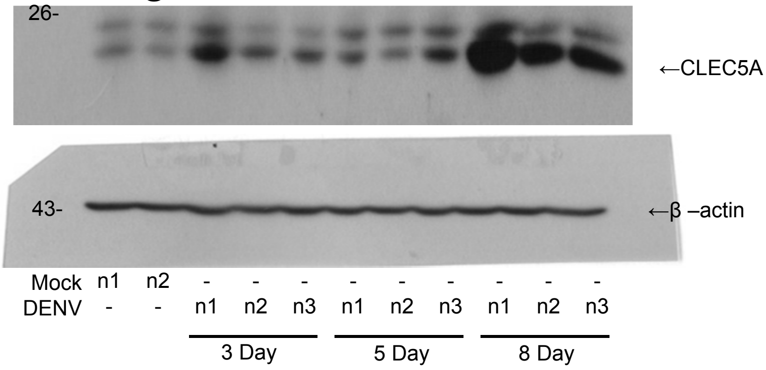

For Figure 6E

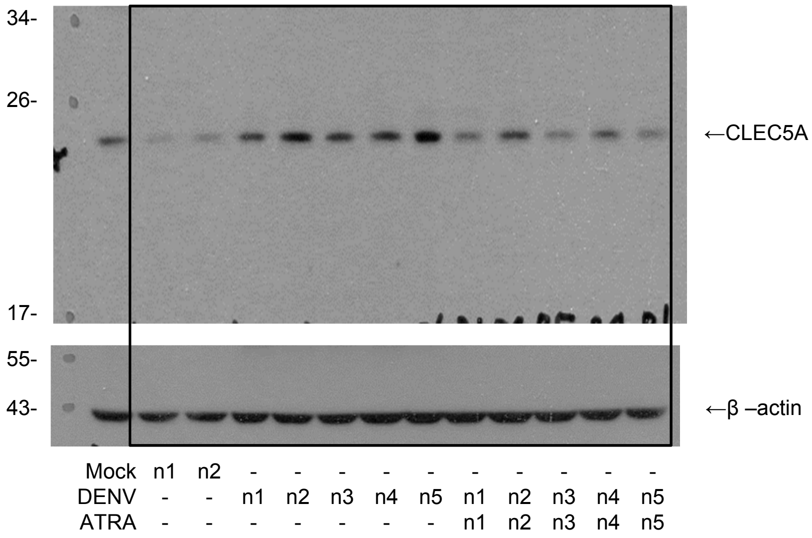

Supplement: Supplementary Information [file srep32000-s1.pdf]
